# A Multidisciplinary Quality Improvement Program to Improve Diabetes Care at a Free Clinic

**DOI:** 10.7759/cureus.36745

**Published:** 2023-03-27

**Authors:** Wade Hopper, Patrick Ruane, JuliSu DiMucci-Ward, Adrienne Z Ables

**Affiliations:** 1 Surgery, Edward Via College of Osteopathic Medicine, Spartanburg, USA; 2 Nutrition, Edward Via College of Osteopathic Medicine, Spartanburg, USA; 3 Family Medicine, St Lukes Free Medical Clinic, Spartanburg, USA

**Keywords:** uninsured, free clinic, type 2 diabetes, dieticians, pharmacists, patient empowerment, multidisciplinary care team

## Abstract

Objective: To evaluate the effectiveness of an intensive, multidisciplinary patient-centered approach involving a pharmacist and a dietician in a population of uninsured free clinic patients with diabetes and hypertension.

Methods: A single-center retrospective chart review of a quality improvement project. All patients had diagnoses of diabetes and hypertension and a most recent hemoglobin A1c ≥ 9.0%. Patients met individually with a pharmacist and a dietician during 6 encounters over 12 months. The pharmacist made medication changes, encouraged lifestyle reflections, and helped patients create and track self-management goals. The dietician helped patients plan strategies for diet and exercise. The primary outcome was a change in mean hemoglobin A1c.

Results: Of 30 enrolled patients, 17 completed three months of treatment, and seven completed 12 months. The 17 patients who completed three months of treatment had the following characteristics: mean age 55.5 years; mean hemoglobin A1c 11.5%; 82% were taking two or more antidiabetic medications; 59% were taking two or more antihypertensive medications. Significant reductions in mean hemoglobin A1c values were observed at three months (-3.4%, P<0.0001) and twelve months (-4.0%, P=0.0156). Reductions in systolic blood pressure were also observed at three months (-6 mmHg, P=0.1060) and twelve months (-17 mmHg, P=0.2188).

Conclusions: Large and significant hemoglobin A1c reductions were observed in free clinic patients with diabetes refractory to traditional medical management. Goal-oriented patient empowerment effectively improves a wide range of patient outcomes in the free clinic setting. Other free clinics can implement this collaborative, multidisciplinary model with access to similar personnel.

## Introduction

In the United States (US), estimates from 2018 indicate that 34.1 million adults aged 18 years or older had a diagnosis of diabetes mellitus (10.2 percent of the US population) and that non-Whites are more likely to have diabetes than Whites [1]. Data from 2013-2016 estimated that 15% of the American population reported current cigarette smoking, 89% were overweight or obese, 38% were physically inactive, 68.4% had a systolic blood pressure > 140 mmHg and or diastolic blood pressure of > 90 mmHg or were on prescription medication for their high blood pressure, and 43.5% had a non-HDL level of > 130 mg/dL [1]. In 2016, 224,000 patients were seen in emergency departments for hyperglycemic crisis and 235,000 for hypoglycemia, while 209,000 and 57,000 were hospitalized, respectively [1]. Finally, the total direct and indirect estimated cost of diagnosed diabetes in the US in 2017 was $327 billion [1].

Diabetes and hypertension disproportionately affect racial minorities and those of low socioeconomic status [2-5]. Free and charitable clinics (FCCs) provide primary care and sometimes specialized medical services to underserved and uninsured patient populations for little or no cost. Over 1,400 FCCs in the United States serve over two million patients annually [6]. A 2010 survey involving 764 FCCs showed that 50% of FCC patients are racial or ethnic minorities and that 92% of FCC patients are uninsured [7].

The quality of care provided at free clinics is largely unknown but is gradually becoming understood. The Institute of Medicine defines quality as the degree to which health services for individuals and populations increase the likelihood of desired health outcomes and are consistent with current professional knowledge [8]. Quality improvement (QI) involves standardizing care processes according to best practice evidence to provide safe, effective, patient-centered, timely, efficient, and equitable [8]. Primary care practices with strong QI cultures continually monitor and assess the efficacy of their care delivery to improve value and patient outcomes, thus fulfilling the triple aim of health care [8]. There are currently no systematic reviews of free clinic patient outcomes. The outcomes literature is characterized by retrospective chart reviews performed largely at student-run free clinics [9-14], although most free clinics are independent entities [7]. Most studies from free clinics are descriptive rather than experimental, and only a few portray multidisciplinary interventions [15-18].

Three studies have described pharmacist management of patients with diabetes in the free clinic setting, with all three studies reporting significant reductions in hemoglobin A1c and two of the studies reporting significant reductions in blood pressure and low-density lipoprotein (LDL) cholesterol [16-18]. Pharmacist-based medication management programs, apart from free clinics, have been associated with significant decreases in hemoglobin A1c and blood pressure [19, 20], improved health outcomes in underserved populations [21], organizational cost savings [20,22], and improved outcomes compared to primary care which does not directly involve pharmacists [23]. Equally important to controlling chronic diseases is medical nutrition therapy (MNT), yet to our knowledge, no study performed at a free clinic has reported biometric outcomes related to MNT. Medical nutrition therapy has significantly improved hemoglobin A1c, systolic blood pressure, weight, and body mass index (BMI) [24]. Previous studies have demonstrated poor nutritional literacy, high rates of food insecurity, and a high prevalence of nutrition-related chronic conditions among free clinic patients [25-27]. Also, low education and income levels are significant risk factors for a poor-quality diet [28]. 

In the inpatient setting, intensive, multidisciplinary care has been shown to effectively lower hemoglobin A1c levels at long-term follow-up of patients with type two diabetes mellitus [29]. However, studies have not evaluated such an approach in the free clinic setting. The free clinic literature contains no data from a multidisciplinary team, including a pharmacist and a dietician. Our quality improvement program aimed to evaluate whether the care of free clinic patients with diabetes and hypertension is improved by an intensive, multidisciplinary patient-centered approach involving a pharmacist and a dietician.

## Materials and methods

Design & Enrollment

We performed a retrospective chart review evaluating the effect of a quality improvement initiative, the Power of Healthy Living program, on biometric outcomes of patients with uncontrolled diabetes and hypertension. The review of the program was approved by the Edward Via College of Osteopathic Medicine Institutional Review Board (R#2022-075). Data were collected at clinic visits over a one-year enrollment period and analyzed through a review of paper charts. Patients seen at St. Luke’s Free Medical Clinic (SLFMC) who were between 18 and 65 years of age and fluent in English were invited to participate in the program. Patient visits were conducted between May 2021 and October 2022. To meet inclusion criteria, patients had to have a diagnosis of type two diabetes mellitus with a documented hemoglobin A1c at or above 9% as well as hypertension treated with at least one antihypertensive medication. Patients who met these criteria were explained the program by SLFMC providers and offered enrollment. Patients were free to exit the program anytime and remain enrolled at SLFMC.

Clinical Setting

St. Luke’s Free Medical Clinic is a 501 (c)(3) nonprofit founded to change the lives of underserved Spartanburg County, South Carolina residents by providing quality and compassionate healthcare. In 2021, SLFMC served 1,600 individuals through 8,090 medical visits [30]. The clinic offers various services, including primary care, mental health counseling, health education, and an on-site pharmacy. To qualify for enrollment, patients must live in Spartanburg County, have a household income at or below 150% of the federal poverty guideline, have no access to any health insurance, and not qualify for Medicare or Medicaid.

Intervention

**Figure 1:**
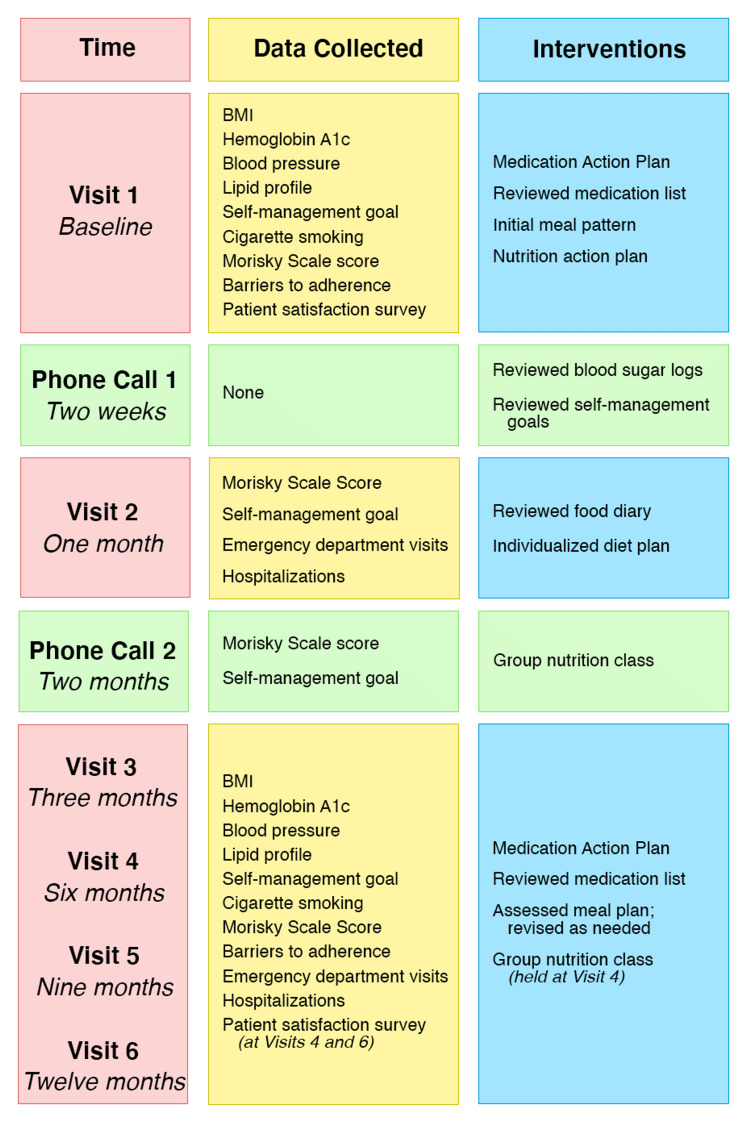
Schedule of Encounters and Procedures A timeline of study encounters is presented alongside the data gathered and interventions performed at each encounter. All clinic visits involved individual meetings with both a pharmacist and a nutritionist. BMI = Body mass index.

The program’s multidisciplinary care team included a primary care provider (PCP), a pharmacist, and a dietitian. The program included four specific components scheduled over one year: pharmacist encounters, dietitian encounters, telephone calls, and a group nutrition class. Patients continued their usual medical visits with their PCP on an as-needed basis. A detailed program timeline is presented in Figure 1.

The pharmacist adjusted medications for diabetes, hypertension, and hyperlipidemia based on laboratory test results and sphygmomanometry readings. Therapeutic goals for each patient were individually determined by American Diabetes Association (ADA) and Joint National Commission 8 (JNC-8) guidelines for diabetes and hypertension, respectively [31,32]. The pharmacist and PCP agreed upon all medication changes. Blood sugar logs were reviewed, and medication reviews and reconciliations were performed during pharmacist encounters, which lasted 30 to 60 minutes in a one-on-one setting. Patients worked with the pharmacist to complete a medication action plan to help them understand their medications and track self-management goals. The pharmacist encouraged patients to reflect upon personal lifestyle choices and to create action plans to achieve self-set health goals. The dietician met with patients to develop individualized meal plans and exercise strategies. The dietary recall was regularly performed, diet plans were individualized, and patients were encouraged to keep diet and blood sugar diaries.

Outcomes

The primary clinical outcomes of the program review were changes in hemoglobin A1c and blood pressure at follow-up intervals compared to baseline. Secondary outcomes included changes in body mass index (BMI), total cholesterol (TC), low-density lipoprotein (LDL) cholesterol, high-density lipoprotein (HDL) cholesterol, triglycerides (TG), cigarette smoking status, and medication adherence. Medication adherence was measured using the Morisky Medication Adherence Scale (MMAS-4), a four-point scale that has been cross-culturally validated and predicts medication nonadherence with roughly 80% sensitivity [33]. Progress toward self-management goals was assessed at each in-person visit. Patient satisfaction was evaluated via brief surveys collected at baseline, six months, and one year. Surveys were administered as in-person self-completed questionnaires, and all survey questions utilized a five-point Likert-type scale. The surveys performed following baseline and six-month visits consisted of four questions and assessed patient satisfaction, patient confidence in their medication and nutrition plans, and perceived helpfulness of the visits. The survey collected at one year assessed additional factors, including whether or not patients would recommend the program to others. Patient demographics of race and ethnicity were reported using language recommended by the Institute of Medicine in concert with the US Office of Management and Budget [34].

Statistical Analysis

Descriptive statistics were used to report patient demographics. The paired t-test was used to calculate significant differences in group means at various intervals compared to the baseline. Data for body mass index, hemoglobin A1c, blood pressure, blood lipids, and medication adherence was collected at baseline and three-month intervals after that. Two-way analysis of variance (ANOVA) was used to compare changes in mean hemoglobin A1c between patients who did and did not meet their self-management goals. Statistical analysis was performed using GraphPad Prism 9® (GraphPad Software, Inc).

## Results

**Figure 2:**
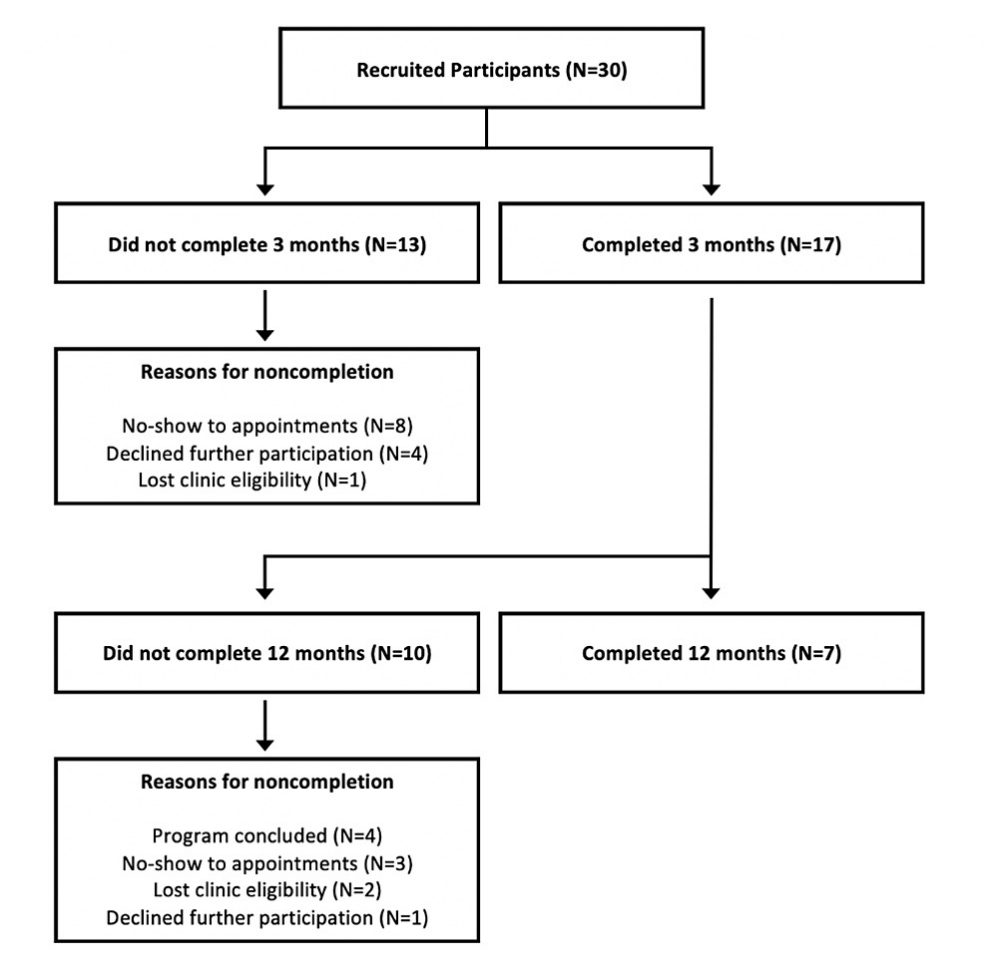
Patient Retention Of 30 enrollees, 17 remained in the program three months from baseline, and seven remained one year from baseline. Missed appointments were the most common reason for participant non-completion.

The program enrolled 30 patients, 17 (57%) of whom completed three months and seven (23%) of whom completed twelve months. The reasons for the 23 program incompletions are shown in Figure 2.

Nearly half of all program incompletions (n=11, 48%) were due to failure to keep scheduled visits. Other reasons included voluntary withdrawal (n=5, 22%), ending of the program timeframe (n=4, 17%), and loss of clinic eligibility (n=3, 13%). Factors influencing patients who voluntarily withdrew included Medicaid eligibility, depression, and caregiver responsibilities. Patient demographics for those who reached three months and one year of enrollment are reported in Table 1.

**Table 1:** Baseline Patient Characteristics Patient demographics and other characteristics present upon enrollment are presented. Values listed are represented as either mean ± standard deviation or as number (percent) as appropriate. MMAS-4 = Morisky Medication Adherence Scale.

Characteristics	Patients who completed three months (n=17)	Patients who completed twelve months (n=7)
Age (Years)	55.5 ± 4.3	55.6 ± 5.9
Sex		
Male	11 (65%)	5 (71%)
Female	6 (35%)	2 (29%)
Race		
White	7 (41%)	4 (57%)
Black	8 (47%)	2 (29%)
Other	2 (12%)	1 (14%)
Ethnicity		
Hispanic	1 (6%)	1 (14%)
Non-Hispanic	16 (94%)	6 (86%)
Smoking Tobacco Use	4 (24%)	0 (0%)
MMAS-4 Score	3.3	3.4
Barriers to Adherence		
Present	10 (59%)	4 (57%)
None present	7 (41%)	3 (43%)
Antidiabetic Medicines, n (%)		
1 medicine	3 (18%)	1 (14%)
2 medicines	5 (29%)	1 (14%)
3 or more medicines	9 (53%)	5 (72%)
Antihypertensive Medicines, n (%)		
1 medicine	7 (41%)	2 (29%)
2 medicines	6 (35%)	3 (42%)
3 or more medicines	4 (24%)	2 (29%)
Statin usage, n (%)	15 (88%)	5 (72%)

At baseline, patients (n=17) had a mean age of 55.5 years, and 11 were male (65%). The twelve-month subgroup (n=7) was similar in composition to the primary cohort regarding age, gender, and ethnicity, although the proportion of Black patients was lower in the subgroup. Baseline hemoglobin A1c means were 11.5% in the primary cohort and 12.5% in the subgroup. All patients took at least one antidiabetic medicine at baseline, and 82% took two or more. The majority of patients were taking insulin. Finally, medication adherence was 3.3 in the primary cohort and 3.4 in the subgroup. Outcomes from patients at three months (n=17) are presented in Table 2.

**Table 2:** Outcomes at Three Months (n=17) Patient outcomes at three months are presented and compared to baseline measures. Values listed are represented as either mean ± standard deviation or proportion (percent) appropriate. BMI = body mass index; SBP = systolic blood pressure; DBP = diastolic blood pressure; TC = total cholesterol; LDL = low-density lipoprotein cholesterol; HDL = high-density lipoprotein cholesterol; TG = triglycerides; MMAS-4 = Morisky Medication Adherence Scale.

Measure	Baseline	Three months	Net change	P
BMI	35.1 ± 8.5	34.7 ± 7.5	-0.4	0.4717
Hemoglobin A1c (%)	11.5 ± 1.7	8.1 ± 1.8	-3.4	< 0.0001
SBP (mmHg)	136 ± 15	130 ± 14	-6	0.1060
DBP (mmHg)	83 ± 16	81 ± 11	-2	0.8082
TC (n=16)	158 ± 55	138 ± 38	-20	0.3591
LDL (n=16)	92 ± 38	82 ± 40	-10	0.3591
HDL (n=16)	42 ± 8	42 ± 11	0	0.7981
TG (n=16)	136 ± 75	127 ± 59	-9	0.5897
Smoking Tobacco Use	4/17 (24%)	2/17 (12%)	-12%	-
MMAS-4 Score	3.3 ± 0.6	3.6 ± 0.5	+0.3	-
Meeting Self-set Goals	-	7/17 (41%)	-	-

A significant 3.4% reduction in hemoglobin A1c from baseline was observed (P<.0001). Reductions were also noted in systolic blood pressure (-6 mmHg), diastolic blood pressure (-2 mmHg), total cholesterol (-20), LDL cholesterol (-10), and triglycerides (-9); however, these results did not reach significance. Of note, 10 of 17 patients (59%) achieved a hemoglobin A1c of < 8.0% at follow-up. Patient outcomes following twelve months of enrollment (n=7) are presented in Table 3.

**Table 3:** Patient Outcomes at Twelve Months (n=7) Patient outcomes at twelve months are presented and compared to baseline measures. Values listed are represented as either mean ± standard deviation or proportion (percent) appropriate. BMI = body mass index; SBP = systolic blood pressure; DBP = diastolic blood pressure; TC = total cholesterol; LDL = low-density lipoprotein cholesterol; HDL = high-density lipoprotein cholesterol; TG = triglycerides; MMAS-4 = Morisky Medication Adherence Scale. † One outlier had a TG level of 584 mg/dl.

Measure	Baseline	Twelve months	Net change	P
BMI	33.9 ± 4.0	33.6 ± 4.8	-0.3	0.7034
Hemoglobin A1c (%)	12.1 ± 2.2	8.1 ± 1.6	-4.0	0.0156
SBP (mmHg)	143 ± 17	126 ± 21	-17	0.2188
DBP (mmHg)	86 ± 19	77 ± 15	-9	0.1327
TC	151 ± 35	131 ± 15	-20	0.2071
LDL	85 ± 33	68 ± 16	-17	0.2562
HDL	41 ± 11	41 ± 7	0	0.8948
TG	176 ± 123	191 ± 180^†^	+15	0.6529
Smoking Tobacco Use	0/7 (0%)	0/7 (0%)	-	-
MMAS-4 Score	3.4 ± 0.8	3.6 ± 0.5	+0.2	-
Meeting Self-set Goals	-	4/7 (57%)	-	-

This subgroup showed a decrease in mean hemoglobin A1c of 4.0% (P=.0156). Reductions in mean values of systolic blood pressure (-17 mmHg), diastolic blood pressure (-9 mmHg), and LDL cholesterol (-17) were observed without statistical significance. Mean triglycerides rose from 176 to 191; however, one outlier had a triglyceride level of 584 mg/dL. Mean HDL cholesterol did not change from baseline in the three-month and twelve-month subgroups.

Patients were asked at baseline to identify personal barriers to better health; these barriers served as discussion points when patients worked with providers to create individualized diet and exercise goals. Ten of 17 patients (59%) endorsed having barriers. Four of the ten patients had somatic barriers, such as partial hemiparesis or chronic leg pain. Cognitive barriers, including forgetfulness, were present in four patients. One patient reported poor exercise tolerance, and another generally cited low motivation as a barrier. Of the initial self-management goals, ten were related to exercise, four were related to diet, two were related to decreased nicotine use, and one was related to medication adherence. Mean hemoglobin A1c reduction at three months was 3.6% among patients who met their initial goals (n=10) compared to 3.0% among patients who did not (n=7); however, two-way ANOVA showed goal achievement did not significantly affect mean A1c reductions at three months (P=.056). Four of seven patients (57%) reported meeting their most recent self-set health goals at the twelve-month visit. Patient satisfaction surveys were collected at intervals of baseline, six months, and twelve months, and the results are listed in Table 4.

**Table 4:** Patient Survey Results Patient satisfaction surveys utilized a five-point Likert-type scale and were collected at baseline, six months, and twelve months. The twelve-month survey utilized additional questions compared to the initial surveys.

	Baseline (n=16)	Six months (n=11)	Twelve months (n=6)
Satisfaction with overall care	4.31	4.45	5.00
Confidence in pharmacist plan	4.75	4.73	4.50
Confidence in dietician plan	4.57 (n=14)	4.64	4.33
Found information helpful	4.75	4.73	-
Would recommend program	-	-	4.67
Satisfied with blood sugar	-	-	4.17
Satisfied with blood pressure	-	-	4.50
Satisfied with weight	-	-	4.00
Feel healthier	-	-	4.50
Equipped with tools to self-manage	-	-	4.00

At baseline, patients were generally confident in their medicine management plans agreed upon with pharmacist counseling and their diet and exercise plans created through collaboration with a dietician. However, two patients still needed to meet with a dietitian by the time of the baseline survey. Results from 11 patients at six months were largely unchanged, with slight increases in overall satisfaction and confidence with their dietitian-guided diet and exercise plans. Overall satisfaction with the program’s care increased with each interval and received a mean rating of five out of a possible five from the six patients enrolled for one year. In terms of specific aspects of health, patients were most satisfied with their blood pressure management and least satisfied with their body weights after completing the program. By the end of the program, patients indicated they felt healthier and would strongly recommend the program to others.

## Discussion

We report hemoglobin A1c improvements, the largest from any multidisciplinary intervention in a free clinic population [16-18]. This information expands public understanding of the quality of care that is possible at FCCs. Over half of all patients reached a goal hemoglobin A1c of < 8.0% by three months of enrollment, even though all patients started at or above a level of 9.0% and the average hemoglobin A1c at baseline was 11.5%. Additionally, mean hemoglobin A1c reductions from the twelve-month subgroup (-4.0%) were only slightly improved compared to improvements made within the initial three months (-3.4%). This suggests that most of the benefits from this intervention occurred within the initial months of the program. The immediate and strong improvements in hemoglobin A1c were primarily due to the intensive, multidisciplinary nature of the methodology, more so than due to any specific medicines, exercise programs, or dietary strategies. A similar phenomenon was observed in a study performed during a short five-day hospitalization involving 61 patients with type two diabetes mellitus who showed a mean hemoglobin A1c reduction of -1.73% (P<.0001) at 12-month follow-up despite the brevity of the initial multidisciplinary treatment [29].

It was observed that patients who achieved self-management goals showed greater mean hemoglobin A1c reductions at three months than those who did not (-3.6% and -3.0%, respectively) and that the effect of goal achievement on hemoglobin A1c was near-significant (P=.056). A significant effect would likely have been observed if the patient population had been larger. Our findings suggest that goal-oriented patient empowerment improves care outcomes in the FCC environment. Further work is needed to explore personal goal setting as a catalyst for change among FCC patients with lifestyle-related chronic diseases.

Although the benefits of pharmacist involvement in the care of FCC patients have been explored [16-18], more is needed about the effectiveness of adding a dietician to the care team in this setting. The prevalence of nutritional counseling services was not assessed by the most recent nationwide survey of FCCs, and this could be a worthy addition to future surveys [7]. Our findings suggest that a pharmacist and nutritionist dietician working in concert can invoke dramatic and immediate changes in hemoglobin A1c in patients with chronic hyperglycemia despite intensive medical management. Lifestyle choices, especially those of diet and exercise, are known to be central risk factors in the development of type two diabetes [35], and pharmacotherapy alone does not address the behavioral drivers of the disease. Unfortunately, the capacity for patients to change is bottlenecked by factors like self-efficacy, motivation, and health literacy. These are modifiable properties, but healthcare providers have limited face-to-face interaction time with patients and often must address complex or multiple concerns per visit. Before the intervention, patients had seen their PCP for visits of standard duration. Our results suggest that dedicated conversation surrounding diet and exercise provided by ancillary specialists can improve FCC patient outcomes and redistribute the burden of teaching traditionally placed on a patient’s PCP. We hypothesize that additional face-to-face time with providers was likely a key driver of lifestyle change in these patients with uncontrolled hyperglycemia refractory to traditional pharmacotherapy.

Overall reductions achieved in blood pressure, total cholesterol, and LDL cholesterol were comparable to those reported by similar studies [16-18], but statistical significance was not observed; this was likely a function of sample size. Reductions in BMI were slight in comparison to improvements in hemoglobin A1c. The observed reductions in BMI likely would have been greater if patients did not require such intensive medical management of their diabetes. Many patients use insulin, associated with weight gain [36]. The majority of patients were on three or more antidiabetic medicines at baseline.

Program satisfaction gradually increased with each visit among seven patients who completed the entire program duration, culminating with a mean rating of five out of five at the final visit. Despite the overall positive reception, patient retention proved to be challenging. Only 57% of enrolled patients completed three months or more of the program. Most patients who withdrew from the study did so voluntarily or missed repeated appointments. Those who withdrew voluntarily cited various life stressors, such as depression and caring for sick family members. Given the abundance of stressors among the FCC patient population, a one-year program presents an onerous commitment. Our data suggest good all-around outcomes and significant improvements in glycemic control are achievable even with a three-month program duration. Future FCC initiatives can consider working with six months or shorter timeframes to mitigate patient withdrawal. Unfortunately, we did not seek feedback from patients who left the program; this is an area of potential improvement for future similar research. A better understanding of factors related to FCC patient dropout is needed to help develop effective solutions. 

Strengths and limitations

We report some of the most robust outcomes data from a free clinic patient population. The program used validated measures for primary clinical outcomes and treatment endpoints by professional consensus guidelines regarding hemoglobin A1c and blood pressure. However, the sample size was small, there was no control group, participants were not randomized, and the causality of outcomes could not be established. Significant reductions in hemoglobin A1c were observed despite these limitations. Reductions in systolic blood pressure, total cholesterol, and LDL cholesterol approached statistical significance, suggesting that a larger sample size could validate the proposed multidisciplinary model across clinical outcomes other than hemoglobin A1c. The data also relied heavily upon self-reporting patients’ self-management goals, medication adherence, and survey responses. We attempted to control self-reporting bias using a validated adherence scale, the four-point Morisky Score. Finally, these results generalize well to FCC patients with uncontrolled diabetes and hypertension. However, the reproducibility of the design is limited to only FCC, which has access to both a pharmacist and a dietician. One study attained similar outcomes and improvements using a pharmacist-led multidisciplinary team with a registered nurse, rather than a dietician, in a health coaching role; this would appear to be a viable alternative to our method for clinics without nutritional specialists [16].

Future directions

The federal government subsidizes medical providers through various initiatives to improve healthcare access, cost, and quality. Most of these initiatives are linked to Medicare and Medicaid payments, disqualifying FCCs from receiving federal financial aid in return for providing quality care. FCCs are instead primarily funded by community goodwill and by non-federal grants. Only 35% of FCCs receive government funding, much of which comes from the state and local levels [7]. As a whole, FCCs may merit future federal investment to strengthen healthcare access and quality for the 11.8% of Americans between 18 and 64 who remain uninsured [37]. Unfortunately, it may prove challenging to incorporate FCCs into legislation due to the great diversity among clinics, and little is known about the quality of care they provide. To understand the quality of care provided by FCCs and to encourage evidence-based practice, the Roadmap to Health Equity consortium has begun to collect and publish clinical outcomes data reported by FCCs nationwide [6]. One purpose of the Roadmap to Health Equity initiative is to better inform political discussion surrounding FCCs by characterizing the sector through outcomes reporting [6]. A secondary benefit of the Roadmap is the standardization of the care provided and the outcomes reported by different FCCs. Improved quality data reporting could also help individual FCCs procure funding, partner with local health systems, and attract volunteer licensed providers [6]. Finally, the current literature holds no systematic reviews of free clinic outcomes; this poses a salient target for future research.

## Conclusions

Our report demonstrates the effectiveness of a pharmacist and dietician working in tandem to provide chronic disease care in a free clinic setting. Large and clinically significant hemoglobin A1c reductions were observed alongside blood pressure, blood lipids, and body mass index reductions. The findings suggest that additional face-to-face time with providers and personalized goal-setting can yield significant therapeutic benefits in patients with diabetes refractory to traditional medical management. Additionally, the clinical involvement of pharmacists, dietitians, and other professionals can ease the workload of primary care providers by redistributing the responsibilities of patient education. Patient retention was challenging beyond three months; however, our results suggest that profound improvements in glycemic control can be attained within three months. As a result, longer timeframes may only sometimes be necessary when performing intensive patient empowerment interventions such as the one we describe. Overall, patients were satisfied with the program and found it helpful. Other free clinics can implement this multidisciplinary quality improvement model with access to similar personnel.
